# Human epidermal growth factor receptor 2 (HER2)-specific chimeric antigen receptor (CAR) for tumor immunotherapy; recent progress

**DOI:** 10.1186/s13287-022-02719-0

**Published:** 2022-01-29

**Authors:** Hendrik Setia Budi, Firdaus Nuri Ahmad, Harun Achmad, Mohammad Javed Ansari, Maria Vladimirovna Mikhailova, Wanich Suksatan, Supat Chupradit, Navid Shomali, Faroogh Marofi

**Affiliations:** 1 Department of Oral Biology, Faculty of Dental Medicine, Universitas Airlangga, Surabaya, 60132 Indonesia; 2 University of Human Development, Sulaymaniyah, Kurdistan Region Iraq; 3 Department of Pediatric Dentistry, Faculty of Dentistry, Hasanuddin University, Makassar, Indonesia; 4 Department of Pharmaceutics, College of Pharmacy, Prince Sattam Bin Abdulaziz University, Al-Kharj, Saudi Arabia; 5 Department of Prosthetic Dentistry, Sechenov First Moscow State Medical University, Moscow, Russia; 6 Faculty of Nursing, HRH Princess Chulabhorn College of Medical Science, Chulabhorn Royal Academy, Bangkok, 10210 Thailand; 7 Department of Occupational Therapy, Faculty of Associated Medical Sciences, Chiang Mai University, Chiang Mai, 50200 Thailand; 8 Immunology Research Center, Tabriz University of Medical Sciences, Tabriz, Iran

**Keywords:** Chimeric antigen receptor (CAR), Human epidermal growth factor receptor 2 (HER2), CAR-T cell, Solid tumors, Tumor microenvironment (TME)

## Abstract

Due to the overexpression or amplification of human epidermal growth factor receptor 2 (HER2) with poor prognosis in a myriad of human tumors, recent studies have focused on HER2-targeted therapies. Deregulation in HER2 signaling pathways is accompanied by sustained tumor cells growth concomitant with their migration and also tumor angiogenesis and metastasis by stimulation of proliferation of a network of blood vessels. A large number of studies have provided clear evidence that the emerging HER2-directed treatments could be the outcome of patients suffering from HER2 positive breast and also gastric/gastroesophageal cancers. Thanks to its great anti-tumor competence, immunotherapy using HER2-specific chimeric antigen receptor (CAR) expressing immune cell has recently attracted increasing attention. Human T cells and also natural killer (NK) cells can largely be found in the tumor microenvironment, mainly contributing to the tumor immune surveillance. Such properties make them perfect candidate for genetically modification to express constructed CARs. Herein, we will describe the potential targets of the HER2 signaling in tumor cells to clarify HER2-mediated tumorigenesis and also discuss recent findings respecting the HER2-specific CAR-expressing immune cells (CAR T and CAR NK cell) for the treatment of HER2-expressing tumors.

## Introduction

Human T cells and also natural killer (NK) cells can be found in the tumor microenvironment (TME) in numerous tumors and arbitrate tumor immune surveillance [1]. T cells mainly support prolonged, antigen-specific, effector, and memory immunological activities [2, 3], and NK cells as the main components of innate immune defense elicit strong anti-tumor functions by secretion of cytolytic granules and inflammatory cytokines as well as chemokines, ensuring the stimulation of both innate and adaptive immune response [4]. The progress of cellular engineering tools has enabled the capability to genetically modify T and also NK cells to express a tumor antigen-specific chimeric antigen receptor (CAR) [5–7]. To date, three CAR T cell-based therapies have acquired FDA approval for treatment of hematological malignancies following achievement of appreciated clinical outcomes by anti-CD19 CAR T cell [8].

In solid tumors, overexpression or amplification of human epidermal growth factor receptor 2 (HER2) concomitant with undesired prognosis has strongly been documented. In fact, amplification or overexpression of HER2 has been detected in about 30% of breast cancers as well as gastric/gastroesophageal cancers and assists as a prognostic and predictive biomarker [9]. HER2 is a member of the epidermal growth factor receptor family demonstrating tyrosine kinase function. HER2 dimerization, in turn, leads to the autophosphorylation of tyrosine residues within the receptor’s cytoplasmic domain and then provokes a myriad of signaling pathways supporting cell proliferation and even tumorigenesis [10]. During the last two decades, the evolution of HER2-redirected treatment has intensely modified the outcome of patients suffering from HER2-positive breast and gastric/gastroesophageal cancers [9]. Although anti-HER2 monoclonal antibodies (mAbs) (e.g., trastuzumab) are presently described as one of the furthermost effective therapeutics in oncology, substantial numbers of patients with HER2-overexpressing tumors, such as breast cancer, show robust resistance to this intervention [11]. Universally, mechanisms for resistance are mainly categorized into four main categories, including hurdles averting trastuzumab binding to HER2, upregulation of HER2 downstream signaling axes, signaling by alternate axes, and also failure to stimulate an immune-mediated mechanism to eliminate transformed cells [12]. Further, trastuzumab-induced cardiotoxicity fences its application in clinic [13]. As a result, development of innovative treatment to target HER2 is of paramount importance. Meanwhile, an efficient method for manufacturing HER2-redirected T or NK cells is the modification of them to show a CAR [14]. CAR-redirected immune cell therapies have superiority over both stem cell transplantation and chemotherapy in terms of safety, and also immunosuppression is not prerequisite in patients receiving CAR T or CAR NK cells. Further, injected CAR T cell can persist in the body long-term, providing durable tumor cell recognition and eradication [15].

Based on the literature, the first HER2-specific CAR was made and described by Dr. Eshhar group in 1993 [16]. Their report evidenced the feasibility of generation of the first-generation HER2 CAR comprising either the zeta (ζ) chain of the TCR/CD3 complex or the gamma (γ) chain of the immunoglobulin receptor FcεRI [16]. They found that engineered CAR T cells could efficiently eradicate HER2-positive target cells and also produce interleukin-2 (IL-2), which usually is applied as a marker to validate the stimulation of CAR T cells [16]. Other studies have shown that direct local administration of the first-generation of HER2-redirected CAR T cells into medulloblastomas in vivo caused the regression in all experimental models [17]. Notwithstanding, robust recurrence observed in all mice signified that first-generation HER2-redirected CAR T cells might be suboptimal for persistence and anti-tumor response because T cells need two signals to become fully activated [17]. Indeed, co-stimulation is urgently prerequisites to facilitate the activation, proliferation, and substantial anti-tumor activity of CAR T cells. After that, much effort has been spent on the manufacture of second- and third-generation CAR T cells, and HER2 has been targeted with these CAR T cells in breast cancer, gastric cancer, sarcoma, glioblastoma, ovarian cancer, and also osteosarcoma [18]. Preliminary studies have signified that the adding of co-stimulation molecules like CD28, 4-1BB, OX-40, ICOS, or CD27 into CAR constructs provides more appreciated therapeutic merits in preclinical and clinical settings [18, 19]. However, the limited achievements driven from T cell immunotherapy mainly in solid tumors highlight the prominence of developing other immunotherapeutics, in particular, NK cell-based treatments [20, 21]. Human NK cells perform as the leading innate immune effector cells versus tumors and are enormously heterogeneous in the TME [22]. Recently, there is rapidly evolving attention in the development of CAR-redirected NK cells for solid tumor therapy. CAR-redirected NK cells have some superiorities over CAR-T cells, such as compromised cytokine release syndrome (CRS) and graft-versus-host disease (GVHD), employing various mechanisms for tumor cell elimination as well as feasibility for ‘off-the-shelf’ manufacturing [23, 24]. Thereby, the advancement of HER2-specific CAR NK cells may support the more desired outcome and prepare a more impressive safety profile as well as efficacy than CAR T cells in some cases.

In the present review, we will firstly discuss the importance of the HER2 in tumor progress and secondly deliver a comprehensive overview about current findings based on the HER2 targeted therapies using CAR expressing immune cells, with special focus on last decade reports.

## CAR redirected cells manufacture

### CARs structure

CARs generally combine the tumor cell recognition competencies of monoclonal antibody variable regions with robust cytotoxic and proliferative capacities of T or NK cells [25, 26]. A typical CAR consists of extracellular antigen recognition domain, a single-chain antibody variable fragment (scFv) that recognize specific antigens in tumors in association with transmembrane and intracellular signaling domains [27, 28]. The intracellular domains include immunoreceptor tyrosine-based activation motifs (ITAMs) existed in the cytoplasmic domains of TCRs as well as other activating receptors. As cited, the first generation of CARs in both CAR-T and CAR-NK consisted of only CD3 as a single activation intracellular signaling domain, which was inefficient in activating immune cells and eradicating tumors. Second- and third-generation CARs consisted of T cell co-stimulatory signaling domains, including CD28, 4-1BB (CD137), ICOS, or OX40 (CD134), in addition to CD3, robustly improving cytotoxicity and proliferative activity and also injected cells in vivo persistence [29–31]. The fourth generations typically use nuclear factor of activated T cell (NFAT) to motivate a promoter related with a cassette containing IL-12 genes [31]. The fifth-generations CARs have been manufactured based on the second generation of CARs, with the addition of a Janus kinase (JAK)-signal transducer and activator of transcription (STAT) activation domain derived from IL-2Rβ. Such domain inspires cell expansion, prohibits terminal differentiation, and bring about the more appropriate persistence [32].

### Immune cell sources

#### T cell

The engineering of autologous peripheral blood (PB) T lymphocytes to selectively identify tumor-associated antigens (TAAs) on the tumor cell surface is currently a well-established method [33]. The ability of T cell receptor (TCR) and CAR therapies by well-established engineering methods is greatly exemplified by the stimulating clinical outcomes achieved with CTA New York esophageal squamous cell carcinoma-1 (NY-ESO-1) TCR and CD19-specific CAR T cells [34, 35]. Such process includes T cell stimulation and transduction to manufacture expanded, genetically modified T cell products [36]. T cells modified to express particular CARs can be originated from Ficoll-purified peripheral blood mononuclear cells (PBMCs), followed by activation with anti-CD3 monoclonal antibody (mAb) in the existence of irradiated allogeneic feeder cells. Then, expanded cells are transduced with a vector encoding CAR [37]. So far, several experimental groups have suggested current good manufacturing practices (cGMP)-compliant large-scale transduction and expansion procedure by either γ-retroviral or lentiviral T cell manufacturing [37]. The appreciated clinical results in the context of engineered T cell therapy can be evidently strengthened by offering potent and available histocompatible T cells [38]. The use of autologous T cells is restricted in patients with chemotherapy or human immunodeficiency virus (HIV)-induced immune deficiency as well as in small infants. Although T cells are simply procured from donors, their application is bargained via the high alloreactive competence [39]. In fact, TCRs expressed on T cell surfaces have a severe tendency to respond toward non-autologous tissues and identify allogeneic human leukocyte antigen (HLA) molecules or even other minor antigens [40]. This inclination underlies graft rejection in transplant recipients and also GVHD occurrence in recipients of donor-isolated T cells [40]. Undoubtedly, preparing allogeneic T cells lacking alloreactive potential is urgently required to obtain a satisfactory risk–benefit ratio. Indeed, the manufacture of the universal allogeneic T cells showing greater anti-cancer impacts is a prerequisite to establish “off-the-shelf” ready-to-use products [41]. During the last two decades, genome-editing tools, including clustered regularly interspaced short palindromic repeat (CRISPR)-Cas9, zinc finger nuclease (ZFN), and also transcription activator-like effector nuclease (TALEN), are being utilized to produce “off-the-shelf” CAR-T cells [42, 43]. Much effort has been spent on the ablation of T cell receptor alpha constant (TRAC or TCR), and also β-2 microglobulin (B2M) by genome-editing technologies to facilitate the production of universal CAR-T cells [44]. These groundbreaking products are now remarked as a novel generation of CAR-T cells, allowing the manufacture of CAR-T cells from allogeneic healthy donors.

#### NK cells

NK cells can be procured from PB and umbilical cord blood (UCB) and also can be established from hematopoietic stem cells (HSCs) or human pluripotent stem cells, ranging from embryonic stem cells (ESCs) to induced pluripotent stem cells (iPSCs) [45, 46]. The clinical scale growth of NK cells enables the achievement of adequate cells for immunotherapy. Importantly, allogeneic NK cells are applied as effector cells due to their inability to induce GVHD, while they promote graft-versus-leukemia (GVL) [47].

Although PB-NK cells are simply procured, their less transduction efficiency concomitantly lower expansion limits their utility [48]. NK cells with higher permissiveness for engineering mainly are generated in large quantities from iPSC [49]. Besides, as shown in the first completed clinical trial based on CAR-NK cells, UCB-NK cells are more readily engineered due to their superior proliferative capability [50]. However, a possible struggle is the fairly immature nature of UCB-NK cells, leading to lower cytolytic function than NK cells derived from PB [51]. Further, there are some differences in the expression pattern of the surface markers between cells isolated from UCB and PB. Indeed, UCB-NK cells show lower levels of CD16, CD2, CD11a, CD18, CD62L, KIRs, DNAM-1, NKG2C, IL-2R, and CD57, and CD8, while higher levels of inhibitory receptor NKG2A than PB-NK cells [52, 53]. Besides, cell lines such as NK-92 provide the fairly limitless source of NK cells for medical use; however, the necessity of their lethal irradiation before administration compromises their retention in the host [54, 55]. As well, the establishment of NK cells from HSC isolated from the BM or UCB prepares other sources for NK cells. The generated cells are largely similar to PB-NK cells and exhibit functionality and the potential to eradicate leukemic cell lines as well as patient’s-derived tumor cells. The HSCs-derived NK cells also produce cytokines upon exposure to several stimuli in vitro and in vivo, whereas display lower rates of inhibitory receptors [56].

## HER2 structure and signaling

The HER proteins are extensively expressed and functionally fundamental in non-hematopoietic tissues [57]. Gene deficient murine models show that HER proteins are crucially implicated in the progress of diverse organ systems, such as the brain, skin, lung, and gastrointestinal tract [58]. The HER family proteins as a well-known type I transmembrane growth factor can regularly trigger intracellular signaling axes in reaction to extracellular signals [57]. Their construction includes three main domains: an extracellular ligand-binding domain, a transmembrane domain along with an intracellular tyrosine kinase domain [59]. The activities of HER proteins are exemplified simply in Caenorhabditis elegans in which signaling is elicited via a solitary ligand and also a solitary receptor [60], and also are demonstrated somewhat more complex in Drosophila in which signaling is mediated through four ligands and a solitary receptor [61]. This axis is more complex in mammalians once the activities of this family are accomplished through about 12 ligands and also four different receptors [62]. We referred readers to some recent superb reviews of HER family signaling and activities [63–65]. Although much is currently understood concerning the molecular basis corresponding to their signaling functions, evident explanations behindhand such multiplicity in HER family structure and signaling is not entirely elucidated. Once ligand connects to HER protein’s extracellular domains, the responding domains undertake dimerization and subsequently transphosphorylates their intracellular domains [66]. The tight interrelations between these phosphorylated tyrosine residues and multiple intracellular signaling proteins result eventually in the triggering a multifaceted downstream second messenger axes [67, 68]. These interrelations finally elicit several biological effects, more importantly, cell proliferation, survival, and also migration [69, 70]. Thereby, a wide spectrum of trials has been conducted or is ongoing to address the safety and efficacy of HER2 targeted therapies using monoclonal antibodies (mAb) in human various tumors (Table 1). The extracellular domain of HER proteins can be usually found in either a closed reserved or an open active form [71]. Binding to responding ligand stimulates a conformational alteration in HER2 extracellular domain, facilitating the induction of active conformation and stimulating their dimerization and resultant transphosphorylation [71]. Growing evidence has shown that partner selection acts as an influential factor in signaling activity among HER proteins. In terms of the catalytic kinase activity, HER2 has superiority over other HER family members and thereby can induce the strongest signaling activities [71, 72]. The evolution of the HER family in mammalian systems has been accompanying by functional differentiation-inducing interdependence but not independent functions, as documented by HER2 and HER3 that are functionally incomplete receptor molecules [73]. In contrast to the other HER family members, HER2 is constitutively in an activated conformation [73]. Besides, unlike the other members, HER3 has no adenosine triphosphate connection with its catalytic domain and is catalytically inactive. Thereby, the signaling activities of HER3 are elicited wholly by the kinase activity of its heterodimeric partners [74]. The fact that even chimeric kinase-active HER3 constructs fail to signal without hetero-partners suggests that HER3 could not form a homodimerization and so is an obligate heterodimerization partner [62]. Irrespective that HER2 and HER3 are necessitating partners, their complex shapes the most active signaling heterodimer of the family and is urgently required for various biologic and developmental procedures [62, 75].

**Table 1:** A summary of clinical trials (Phases 2, 3, and 4) based on the human epidermal growth factor receptor 2 (HER2)-targeted therapies using specific anti-bodies in HER2 positive tumor cells registered in ClinicalTrails.gov (September 2021)

Condition	Drug	Study phase	Status	Participant number	Location	NCT number
Advanced Breast Cancer	GB221	3	Recruiting	338	China	NCT04164615
Advanced Breast Cancer	Trastuzumab	2	Recruiting	16	USA	NCT04329065
Breast Cancer	Trastuzumab	2	Not yet recruiting	59	China	NCT04034823
Breast Cancer	Bevacizumab	2	Completed	50	USA	NCT00095706
Breast Cancer Gastric Cancer Gastroesophageal Junction Cancer	ZW25	2	Recruiting	50	China S. Korea Taiwan	NCT04276493
Breast Cancer	GB221	2	Recruiting	132	China	NCT04170595
Breast Cancer	RC48-ADC	2/3	Recruiting	301	China	NCT03500380
Breast Cancer	Trastuzumab	2	Completed	55	USA	NCT00019812
Colorectal Cancer	Trastuzumab	2	Completed	32	USA	NCT00003995
Urothelial Carcinoma Advanced Cancer	RC48-ADC	2	Completed	43	China	NCT03507166
Advanced Solid Tumors	Ertumaxomab	2	Terminated	14	Germany	NCT01569412
Breast Cancer	Trastuzumab	2	Completed	50	USA	NCT00003539
Endometrial Cancer	SYD985	2	Recruiting	60	International	NCT04205630
Gastric Cancer	Trastuzumab	2	Recruiting	52	China	NCT04661150
Breast Cancer	Trastuzumab	2	Completed	37	USA	NCT00006228
Breast Cancer	Trastuzumab	2	Completed	200	USA	NCT00003992
Breast Cancer	Trastuzumab	2	Recruiting	90	France	NCT03571633
Non-small-cell lung carcinoma (NSCLC)	Zenocutuzumab	2	Recruiting	250	International	NCT02912949
Breast Cancer	Pertuzumab	2	Completed	70	International	NCT02491892
Gastric Cancer	HLX22	2	Not yet recruiting	150	China	NCT04908813
Breast Cancer	Pertuzumab Trastuzumab	2	Completed	37	USA	NCT00301899
Breast Cancer	MRG002	2	Recruiting	60	China	NCT04924699
Urothelial Carcinoma	MRG002	2	Recruiting	58	China	NCT04839510
Metastatic Biliary Tract Cancer	MRG002	2	Recruiting	86	China	NCT04837508
Biliary Tract Cancers	ZW25	2	Recruiting	100	USA	NCT04466891
Gastrointestinal Cancer	ZW25	2	Recruiting	362	USA Canada S. Korea	NCT03929666
Breast Cancer	ARX788	2	Recruiting	200	USA Australia	NCT04829604
Colorectal Neoplasm	DS-8201a	2	Completed	70	International	NCT03384940
Advanced Solid Tumors	MRG002	2	Recruiting	152	China	NCT04492488

*NA* not applicable

## HER2 overexpression in human cancer

The significance of pre-clinical information to human cancers is sustained by a large body of clinical outcomes. The overexpression of the HER2 protein by gene amplification or transcriptional deregulation is identified in about 30% of breast and ovarian cancers, largely contributing to tumor progression and metastasis [76]. Breast tumors can involve about 50 copies of the HER2 gene and up to 40- to the 100-fold increase in HER2 protein expression leading to the expression of approximately 2 × 10^6^ receptors on the tumor cell surface [62]. Interestingly, analysis has provided strong evidence that HER2 amplification is accompanied by several undesired biological processes and also could be pronounced as an early event in human breast tumorigenesis [77]. Nonetheless, enhancement in HER2 expression is detected in nearly half of all in situ ductal carcinomas without any invasiveness [62]. Moreover, HER2 overexpression has been found as an oncogenic driver and plausible therapeutic target in pulmonary cancers [78]. A study of the tumor samples from 175 patients with pulmonary adenocarcinomas for examination of the existence of HER2 amplification and mutation and HER2 protein overexpression revealed that HER2 amplification was seen in 3%, HER2 mutation was detected in 4%, and finally, HER2 overexpression was found about 14% [78]. These findings implied that HER2 mutations are not allied with HER2 amplification, so signifying a separate entity and therapeutic target. As well, another report evinced that non-small-cell lung carcinoma (NSCLC) tumor overexpressing both EGFR and HER2 might exhibit higher sensitivity to EGFR tyrosine kinase inhibitor (TKI) compared to EGFR but not HER2 overexpressing tumors [79]. Accordingly, the determination of EGFR and HER2 protein expression levels may possess a prognostic worth in non-small cell lung cancer (NSCLC) [79]. Besides, studies on 37,992 patient samples for assessment of HER2 expression levels demonstrated that HER2 protein overexpression was seen in 2.7% of samples predominantly in cancers of epithelial origin [80], while overexpression of HER2 protein was detected in 7–34% of gastric cancers [81]. As well, HER2 gene amplification was seen mainly in noninvasive gastric carcinoma and took place throughout the early steps of gastric cancer and also exposed heterogeneity in several cases [81, 82]. Assessment of the HER2-amplification/overexpression in stage II–III and IV colorectal cancer (CRC) patients also displayed that 2.2% stage IV and 1.3% stage II–III tumors show overexpression of HER2 protein. Of the HER2-overexpressing cases, about 96% stage IV tumors and 84% stage II–III tumors showed HER2 amplification [83]. Further, HER2 overexpression is closely linked to the patient's survival in pancreatic carcinoma [84, 85]. Meanwhile, Komoto et al. [86] found that HER2 could be found in about 61% of patients with pancreatic carcinoma and led finally to the toughly shorter survival times.

These consequences inspire further examination of treatments utilizing new molecular targeting agents or cellular products against HER2 protein or HER2-positive cells to improve the survival of patients suffering from various tumors.

## Transcriptional targets of HER2

**Fig. 1:**
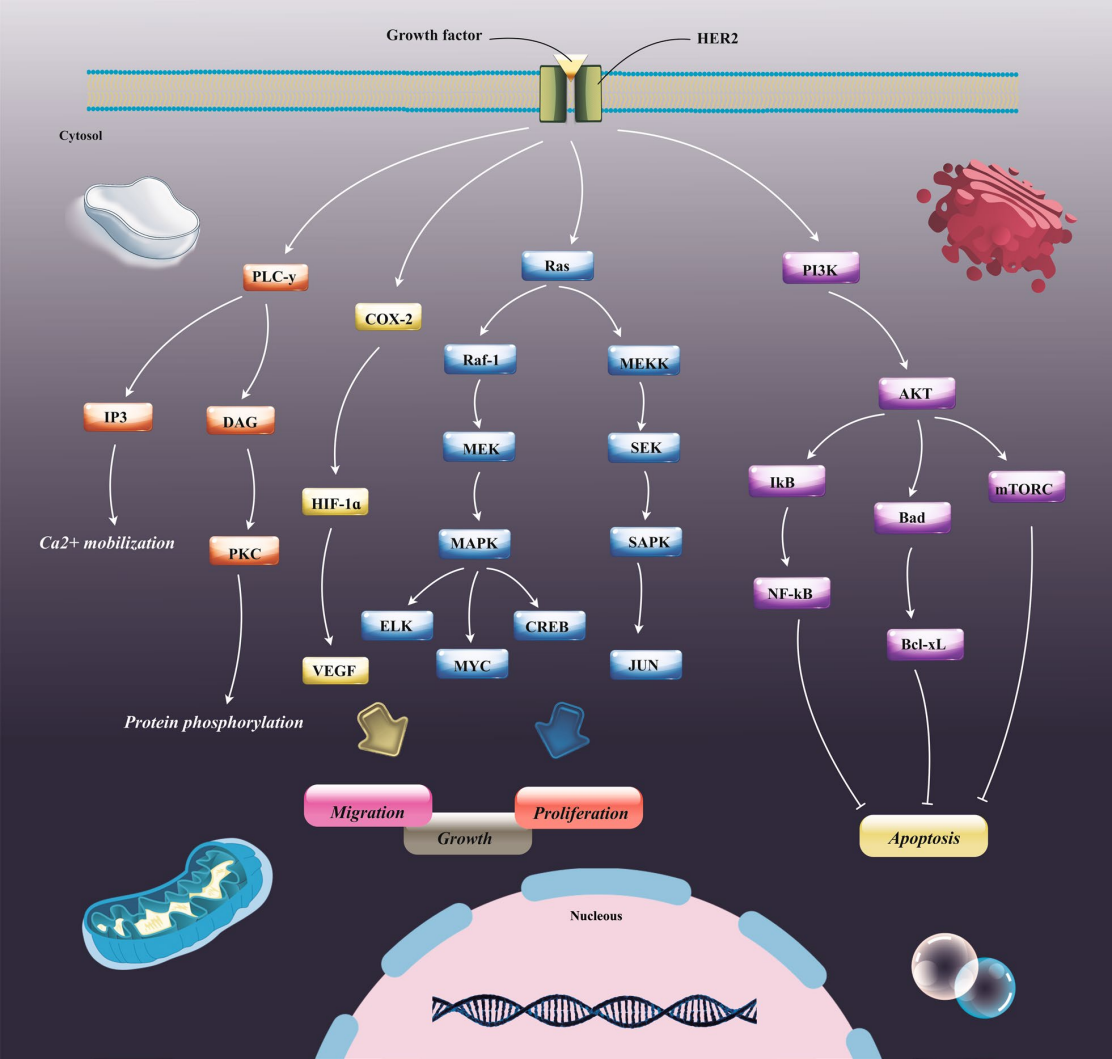
HER2 signaling pathway. HER2 and other EGFR family members as receptor tyrosine kinases positioned on the cell membrane can responds to various ligands. Phosphorylation of the tyrosine kinase domain in the cytoplasm instigates subsequent oncogenic signaling axes, such as PI3K/AKT pathway and Ras/MAPK pathway. Human epidermal growth factor receptor 2 (HER2), Phosphoinositide 3-kinase (PI3K), Mitogen-activated protein kinase (MAPK), Mammalian target of rapamycin complex (mTORC), BCL2 associated agonist of cell death (BAD), Nuclear factor kappa-light-chain-enhancer of activated B cells (NF-κB), inhibitor of nuclear factor kappa (IκBα), B-cell lymphoma-extra-large (Bcl-xL), Mitogen-activated protein kinase (MAP2K or MEK), MEK kinase (MEKK), Stress activated protein kinases (SAPKs), cAMP-response element binding protein (CREB), Cyclooxygenase-2 (COX-2), Hypoxia-inducible factor-1 (HIF-1), Protein kinase C (PKC), Phospholipase C gamma (PLCγ), Diacyl glycerol (DAG), Inositol 1,4,5-trisphosphate (IP3), Vascular endothelial growth factor (VEGF), Epidermal growth factor receptor (EGFR)

When HER signaling pathways are activated, induction of subsequent signaling axes may result in growth and spread of cancer cells (Fig. 1). It has previously been found that HER2 directly adjusts cyclooxygenase 2 (COX-2) expression by transcriptional induction [87, 88]. Subbaramaiah et al. have described that the inducible prostaglandin synthase COX-2 is greatly overexpressed in HER2-positive breast cancers [89]. Importantly, other reports have outlined that mice with COX-2 deficiencies mainly show abrogated mouse mammary tumor virus (MMTV)-neu-stimulated mammary tumorigenesis [89]. Correspondingly, study of the possible relation between the COX-2 and HER-2 in colorectal cancer (CRC) indicated that in addition to the presence of a robust association between COX-2 and HER-2 expression in CRC, both of them are an important player in the invasion and metastasis of CRC [90]. In addition, HER2 elicits the expression of the chemokine receptor C-X-C motif chemokine receptor 4 (CXCR4) in transfection models and also up-regulated CXCR4 expression is typically found in breast cancers with high rates of HER2 expression [91]. Thanks to that CXCR4/CXCL12 chemokine interaction facilitates tumor cell proliferation and leads to the development of distant metastases, it appears that metastasis of breast cancers may mainly arise from the overexpression of chemokine receptors. This fact supports the premise that the prometastatic features of HER2-overexpressing tumors are likely mediated by the elevated expression of relevant chemokine receptors [92]. This hypothesis is validated by other findings, implying that the HER2-mediated migratory potential of tumor cells could be repressed by anti-CXCR4 antibodies [93]; on the other hand, HER2 suppresses ligand-mediated CXCR4 degradation and thereby potentiates the responding signaling [93]. Another study has suggested that impaired primary tumor development and metastases in orthotopic esophageal carcinoma models upon direct inhibition of HER2 might be attributable to resultant suppression of CXCR4 expression [94]. In contrast, analysis of 148 ovarian tumor samples showed that there was no correlation between the expression of CXCR4 and HER2 overexpression; however, overexpression of HER2 had a strong association with overall survival in ovarian cancer patients [95]. In addition, HER2-overexpressing have been implicated in up-regulation of E26 transformation-specific (ETS) transcription factors in breast tumors [96]. HER2 particularly sustains the bimodal stimulation of the ETS transcription factor ER81, which in turn, enables the expression of the telomere’s catalytic subunit [96]. HER2 and ER81 can cause a synergistic enrichment in the transcriptional activation of telomerase reverse transcriptase (hTERT), which accompanied with telomerase RNA component shapes the most pivotal unit of the telomerase complex [97, 98]. Another study also has shown that the phosphoinositide 3-kinases (PI3K) PI3K/protein kinase B (PKB or Akt) pathway is a critical downstream signaling pathway of HER2 in gastric cancer patients [99]. It seems that HER2 down-regulate phosphate and tensin homolog (PTEN) expression as well as activation in gastric cancer, leading to the tumorigenesis [99]. As well, Kallergi et al. showed that HER2 is expressed on circulating tumor cells of 38% and 50% breast cancer patients. Similarly, they verified existence of association between HER2 expression and phospho-PI3K and phospho-Akt expression levels in circulating tumor cells [100]. HER2 activates PI3K/Aktsignaling independent of HER3 in human tumors [101]. Second messenger pathways, comprising Ras or Grb2, are found to be responsible for HER3-independent activation of the PI3K/Akt axis [101]. This fact that small-molecule inhibitors of Akt could circumvent tumor cells’ resistance to anti-HER2 therapies confreres the central roles of PI3K/Akt axis in HER2-mediated tumorigenesis [102]. Also, HER2 overexpression leads eventually to the up-regulation of hypoxia-inducible factor 1α (HIF-1α) by Akt activation. The HIF-1α acts typically as the efficient positive regulator of vascular endothelial growth factor (VEGF) and fibronectin receptors expression [103, 104]. Likewise, Jarman and coworkers showed that HER2 overexpression in MCF7 cells caused enhancement in HIF-2α but not HIF-1α expression in normoxia and an also promoted HIF-2α expression in hypoxia [105]. Moreover, up-regulation of MMP-2 and MMP-9, and also activation of the nuclear factor (NF)-κB anti-apoptotic pathway may arise from HER2 overexpression, as shown strongly in breast and gastric cancers [106, 107]. Similarly, HER2 and proto-oncogenes MYC are commonly coamplified in breast cancer, correlated with aggressive clinical behavior and undesired outcome because of the eliciting stem-like phenotype [108]. Deregulated MYC appears that is not tumorigenic lonely, but coexpression with HER2 supports the amplified MYC Ser62 phosphorylation and accelerated tumorigenesis [109, 110]. As well, tyrosine phosphatase protein tyrosine phosphatase alpha (PTPα/PTPRA), a well-known negative regulator of tumor cell apoptosis, contributes to HER2-induced breast tumor onset and maintenance, as evidenced by Meyer et al. reports [111]. Notably, there is also some evidence indicating that HER2 could stimulate expression of prostate-specific antigen (PSA) by up-regulation of MAP kinase pathway in prostate cancer cells [112]. Hence, due to the diversity of pathways affected by HER protein, it is not surprising that HER2-targeted therapies have extremely ameliorated survival outcomes for HER2-positive cancer patients.

## HER2-specific CARs in human tumor therapy

As described, a large number of studies have investigated the therapeutic potential of HER2- CAR T cell (Table 2) and also HER2-CAR NK cell (Table 3)-based therapies in a diversity of human tumors.

**Table 2:** A summary of recent reports based on CAR T cell therapies to target EGFR positive tumor cells, in particular, HER2-overexpressing tumor cells

Cancer	Target Ag	Study model	Results	References
Triple-negative breast cancer (TNBC)	HER1 HER2	In vitro In vivo	Induction of HS578T, MDA-MB-468, MDA-MB-231, and MCF-7 cell line elimination in vitro Inhibition of the development of xenograft TNBC tumors in mice	[156, 157]
Non-small-cell lung carcinoma (NSCLC)	NA	In vitro In vivo	Selective eradication of A549, H23, and H1299 cell lines in vitro Abrogated tumor progress of xenografts NSCLC in mice	[158]
Glioblastoma	EGFRvIII	In vitro In vivo	Stimulation of non-significant anti-tumor response against the heterogeneous EGFRvIII expressing tumors	[159]
Mammary cancer	HER2	In vivo	PTPN2 ablation in T cells improved anti‐tumor response and CAR T‐cell efficacy in mice xenografts	[160]
Glioblastoma	EGFRvIII	In vivo	Selective elimination of EGFR-expressing tumor cells in U87 glioma cell bearing NSG mice	[161]
Melanoma	HER2	In vitro In vivo	Elimination of SK-MEL-1 cell line in vitro Profound or complete abrogation of tumor progress in xenograft tumors in mice	[162]
Glioblastoma	EGFRvIII	In vivo	Stimulation of antigen loss and also adaptive resistance in patients with recurrent glioblastoma upon infusion of a single dose of EGFRvIII-specific CAR T cells	[163]
Glioblastoma	HER2	In vitro In vivo	Tandem CAR T cells affecting HER2 and IL13Rα2 attenuated tumor antigen escape in vitro and in vivo	[164]
Squamous cell carcinoma (SCC)	NA	In vitro	Selective eradication of FaDu cells in vitro	[165]
Pancreatic ductal adenocarcinoma (PDA)	HER2	In vivo	Switchable CAR-T cells trailed by injection of a Fab-based switch directed against HER2 showed higher efficacy against the advanced pancreatic tumors in NSG mice	[121]
Glioblastoma	EGFRvIII	In vivo	Intra-tumoral IL-12 delivery boosted the efficacy of CAR-T cell immunotherapy in tumor cell bearing C57Bl/6 mice	[166]
Glioblastoma	EGFRvIII	In vitro In vivo	Notable antitumor impacts of EGFRvIII-specific CAR-T cell therapy along with PD-1 checkpoint blockade in glioblastoma cells	[167, 168]
NSCLC	NA	In vivo	Verification of the safety and feasibility of EGFR-specific CART cell against EGFR-positive NSCLC	[168]
Glioblastoma	EGFRvIII	In vivo	PD-1 ablation using CRISPR-Cas9 led to the ameliorated anti-tumor function of EGFRvIII-specific CAR T cells in tumor cell bearing mice	[169, 170]
Ovarian cancer	HER2	In vivo	Low-affinity (LA)-CARTs demonstrated lower liver injury and less systemic rates of IFN-γ than high-affinity (HA)-CARTs in xenograft mice	[171]
NSCLC	HER2	In vivo	Docetaxel (DOC) improved the infiltration of HER2-CAR T cells to tumor area in the NSCLC mice model	[172]
Various tumors	HER2	In vitro In vivo	HA-CAR T cells produced higher rates of IFN-γ and IL-2 than LA-CAR T cells	[173]
Head and neck squamous cell carcinomas (HNSCC)	NA	In vitro In vivo	EGFR-specific CAR T cells considerably reserved the proliferation of radio-resistant Cal33 tumor cells in vitro and mice xenografts	[174]
Glioblastoma	EGFRvIII	In vivo	EGFRvIII-specific CAR T cells reduced tumor development in mice xenografts	[175]
Various tumors	HER2	In vitro	HER2Bi-armed CART19 showed selective cytotoxicity versus various HER2+/EGFR+/CD19- breast, pancreatic, ovarian, prostate, and lung cancer cell lines	[123]
NSCLC	EGFRvIII	In vitro In vivo	Efficient antitumor function versus A549 cell line in vitro and in vivo	[176]
Various tumors	HER2	In vivo	Restoring the antitumor impacts of HER2-specific CAR T cells by delaying epithelial-mesenchymal transition in tumor cells	[177]
Sarcoma	HER2	In vivo	Verification of the safety and efficacy of HER2-specific CAR T cells in patients with sarcoma for 6 weeks lacking apparent toxicities	[178]
Hepatocellular carcinoma (HCC)	EGFRvIII	In vitro In vivo	EGFRvIII-specific CAR T cells established by piggyBac transposon displayed robust growth suppression versus HCC cell lines in vitro and in vivo	[179]
NSCLC	NA	In vivo	Verification of the safety and efficacy of EGFR-specific CAR T cells against EGFR-positive NSCLC patients	[180]
Ovarian cancer	HER2	In vitro	Inhibition of tumor cells proliferation by HER2-specific CAR-T cells	[181]
Colorectal cancer (CRC)	HER2	In vivo	Regression or elimination of CRC xenograft in tumor cell bearing NOD-NPG mice	[139]
Breast cancer	HER2	In vivo	A small population of HER2-specific CAR T cells could stimulate an anti-tumor response against breast cancer xenografts	[182]
Glioblastoma	EGFRvIII	In vivo	EGFRvIII-specific CAR T cells showed no clinically significant effect in patients with glioblastoma	[183]
TNBC	NA	In vitro In vivo	Combination therapy with THZ1 and EGFR CAR T cells restored immune resistance, reduced tumor proliferation, and also metastasis in TNBC xenografts	[184]
HCC	NA	In vitro In vivo	EGFR-specific CAR T cells demonstrated poor proliferation activity and cytotoxicity versus HCC cells	[185]
Gastric cancer	HER2	In vitro In vivo	Robust cytotoxicity against HER2-positive gastric cancer cells	[141]

*PD1* programmed cell death protein 1, *CRISPR* clustered regularly interspaced short palindromic repeats, *IFN-γ* interferon gamma, *NA* not applicable

**Table 3:** A summary of recent reports based on CAR NK cell therapies to target EGFR positive tumor cells, in particular, HER2-overexpressing tumor cells

Cancer	Target Ag	Study model	Results	References
Glioblastoma	EGFRvIII	In vitro In vivo	Elimination of glioblastoma cell lines by IFN-γ production Robust regression of tumor progress and considerably extended overall survival rate of the tumor-bearing mice upon intracranial infusion of NK-92-EGFR-CAR cells	[186, 187]
Breast cancer	NA	In vitro In vivo	EGFR-CAR NK-92 cells and primary NK cells showed improved cytotoxicity and IFN-γ generation against MDA-MB-231, MDA-MB-468, and MCF-7 cell lines Combination therapy with EGFR-CAR NK-92 cells and oHSV-1 led to the more effective eradication of MDA-MB-231 tumor cells in mice xenografts	[188]
Glioblastoma	EGFRvIII	In vitro	EGFRvIII -CAR NK-92 cells suppressed the proliferation and induced apoptosis in glioblastoma cell lines	[189]
Glioblastoma	EGFRvIII	In vivo	EGFRvIII -CAR NK-92 cells modified to overexpress CXCR4 ameliorated immunotherapy of CXCL12/SDF-1α-producing U87-MG glioblastoma cell bearing mice	[190]
Breast cancer Renal cancer	HER2	In vitro In vivo	HER2-CAR NK-92 cells robustly lysed HER2-expressing tumor cells in vitro Selective detection and elimination of tumor cells in orthotopic breast carcinoma xenografts by HER2-CAR NK-92 cells Attenuation of pulmonary metastasis in renal cell carcinoma xenografts	[191]
Triple-negative breast cancer (TNBC)	NA	In vitro In vivo	EGFR-CAR NK cells lysed EGFR-expressing TNBC cells in vitro EGFR-CAR NK cells suppressed both cell line-derived xenografts and patient-derived xenografts tumors in xenografts	[192]
Real cell carcinoma (RCC)	NA	In vitro In vivo	Cabozantinib boosted the EGFR and diminished PD-L1 expression in RCC cells and eventually restored the cytotoxicity of EGFR-CAR-NK-92 cells versus the RCC cells in vitro	[193]
Glioblastoma	EGFRvIII	In vitro	EGFRvIII-CAR NK cells showed no significant effect on tumor progression	[194]
TNBC	HER2	In vitro In vivo	HER2-CAR NK-92 cells improved the lysis of HER2‑expressing MDA-MB-453 and SKBr3 cell lines in vitro HER2-CAR NK-92 cells selectively decreased tumor volume and pulmonary metastasis of nude mice bearing established MDA-MB-453 cells, and also elevated the overall survival rate of the xenografts	[195]
TNBC Ovarian cancer	HER2	In vitro	HER2-CAR NK-92 cells selectively and proficiently eliminated established and primary HER2-expressing tumor cells in vitro	[196]
Glioblastoma	NA	In vivo	Combination therapy with EGFR-CAR NK cells oncolytic virus expressing IL-15/IL-15Rα abrogated the proliferation and considerably improved the survival rate of mice xenografts possibly by augmented intracranial recruitment and activation of NK and CD8+ T cells	[197]

*oHSV* oncolytic herpes simplex virus, *PD1* programmed cell death protein 1, *NA* not applicable

### Glioblastoma multiforme (GBM)

The GBM is the most aggressive and incurable human primary brain tumor. Immunotherapies currently have shown the competence to affect GBM stem cells, which mainly demonstrate robust resistance to conventional therapies. Concerning reports, HER2-specific CAR T cells can be created from GBM patients to target HER2-expressing GBMs and their CD133-expressing stem cells [113]. HER2-specific CAR T cells are usually established by transduction with a retroviral vector encoding anti-HER2 CAR [113]. Anti-HER2 CAR T cells could produce remarkable levels of IFN-γ and IL-2 upon detecting HER2-expressing autologous GBM cells, ultimately leading to the persistent regression of autologous GBM xenografts. Substantially, HER2-CAR T cells could eliminate CD133-positive and CD133-negative cells achieved from primary HER2-expressing GBMs, but not HER2-negative tumor cells [113]. These observations signify that the adoptive transfer of HER2-specific CAR T cells can be a favorable immunotherapeutic tactic for GBM [113]. HER2-redirected CAR T cells could also exhibit strong cytotoxicity versus human glioblastoma U251 cells in vitro [114]. As well, the efficacy of anti‑HER2 CAR T cell therapy could be ameliorated by combination therapy with PD1 blockade [114]. Blocking PD-1-mediated immune suppression strongly enhances the stimulation of CAR T cells and thus, demonstrates a great therapeutic capacity for hindering the development of malignant glioblastoma [114]. Besides, trivalent T-cell product, known as universal tricistronic transgene (UCAR) T cells, which armed with three CAR molecules detecting HER2, IL-13 receptor subunit alpha-2 (IL13Rα2), and ephrin-A2 (EphA2), has showed promising outcomes for GBM therapy [115]. Meanwhile, co-targeting HER2, IL13Rα2, and EphA2 revealed the strong capability to defeat interpatient inconsistency by a propensity to capture about 100% of tumor cells [115]. UCAR T cells also could fashion vigorous immune synapses with tumor targets, created more polarized microtubule forming centers, and concurrently elicited better cytotoxicity and cytokine generation than both monospecific and bispecific CAR T cells [115]. Similarly, T cells co-expressing HER2 and IL-13Rα2 specific CARs have shown heightened antitumor activity in the glioma orthotopic xenogeneic murine model [116, 117]. Besides, an open-label phase 1 dose-escalation study on 17 patients with advanced HER2-positive showed that 1 or more infusions of autologous HER2-CAR T cells were well tolerated without any treatment-related serious event [118]. Of 16 evaluable participants, 1 experienced a partial reaction for more than 9 months, 7 experienced steady disorders for 8 weeks to 29 months, and 8 experienced disease progresses upon intervention. Observation universally evidenced the safe and potent efficacy for patients suffering from GBM [118].

As cited, NK cells can also be engineered to show CARs that identify TAA and trigger selective detection and specific lysis of cancer cells. In this regard, HER2-specific CAR NK-92/5.28.z cells strongly eradicated GBM tumor in NOD-SCID IL2Rγ (null) (NSG) mice and C57BL/6 mice and led to the improved overall survival rate of treated mice [119]. In contrast to untargeted NK-92 cells, CAR NK-92/5.28.z cells eliminated all HER-expressing established and primary GBM cells in vitro [119]. Interestingly, in immunocompetent mice, NK-92/5.28.z cell’s local administration caused cures of transplanted syngeneic GBM in 4 of 5 mice with subcutaneous tumors and 5 of 8 mice bearing intracranial tumors, justifying assessment of this method for HER-positive GBM patients [119].

### Pancreatic cancer

Pancreatic cancer is a deteriorating gastrointestinal cancer characterized by late diagnosis, limited treatment success, and poor prognosis. Exocrine tumors account for 95% of pancreatic cancers, and the most shared pathological kind is pancreatic ductal adenocarcinoma (PDAC) [120]. Evaluating the efficacy of conventional and switchable CAR T cells to affect the HER2-positive PDAC using patient-derived xenograft (PDX) models established from patients with stage IV PDAC revealed that CAR T cell therapy could lead to the complete remission in difficult-to-treat patient-derived tumors [121]. It was speculated that switchable HER2-redirected CAR T cells are as effective as conventional HER2-redirected CAR T cells in vivo [121]. Another report has shown that HER2-specific CD8+CD161+ CAR T cells could eradicate HER2-expressing PDAC cells faster and with greater efficiency than HER2-specific CD8+CD161− CAR T cells [122]. Further, CD8+CD161+ CAR T cells could stimulate in vivo antitumor efficacy in xenograft models of HER2-expressing PDCA cells, with raised expression of granzymes and abridged expression of exhaustion markers [122]. Besides, Thakur et al. [123] employed anti-CD19 CART armed with anti-CD3 (OKT3) × anti-HER2 bispecific antibodies (HER2Bi) or anti-CD3 (OKT3) × anti-EGFR bispecific antibodies (EGFRBi) to address the cytotoxicity against HER2 or EGFR positive cancer cell lines. They observed specific cytotoxicity mediated by anti-CD19 CAR T cells armed with HER2Bi or EGFRBi versus breast, pancreatic, ovarian, prostate, and lung cancer cell lines [123]. HER2Bi- or EGFRBi-armed anti-CD19 CAR T cells displayed specific cytotoxicity toward multiple HER2+/EGFR+/CD19- tumor targets in long-term serial killing assays. As well, anti-CD19 CAR T cells presented ameliorated survival along with augmented resistance to depletion following repetitive exposure to tumor cells [123]. On the other hand, assessment of the safety, feasibility, and efficacy of HER2-redirected CAR T cells in patients with advanced biliary tract cancers (BTCs) and pancreatic cancers during a phase I clinical trial (NCT01935843) exhibited promising signs of clinical safety, feasibility and also activity [124]. Meanwhile, administration of HER2-specific CAR T cells preconditioned with nanoparticle albumin-bound paclitaxel (nab-paclitaxel) and cyclophosphamide caused mild-to-moderate fatigue, nausea/vomiting, myalgia/arthralgia, and lymphopenia. While in terms of efficacy, about 50% of participants exhibited a partial response to intervention or experienced stable disease [124].

### Breast cancer

Advances in HER2-targeted treatments have enhanced the survival of patients with HER2-positive breast cancer [125]. Yet, breast cancer’s metastasis to the brain remains a substantial clinical problem, while it can be circumvented with HER2-redirected CAR immunotherapy [126]. For example, Seyedmirzaei and his coworkers found that single dose of HER2-specific CAR T cells could eliminate tumors and improve long-term survival in trastuzumab-resistant breast tumor cell-bearing mice. It appears that CAR T cells could also penetrate the tumor matrix, which usually referred as a barrier for antibodies [126]. There is other indication demonstrating that even a small quantity of HER2-redirected CAR T cell could trigger a remarkable anti-tumor activity versus antibody-resistant xenograft [127]. Nonetheless, the optimizing CAR design for solid tumors is of paramount importance because of lacking truly restricted antigen expression and possible safety issues with the "on-target off-tumor" function [128]. Accordingly, Priceman et al. optimized HER2-redirected CAR T cells to obstruct breast-to-brain metastases. HER2-CAR constructs were comprised of either CD28 or 4-1BB domains and demonstrated functional activity in vitro, as documented by the assessment of cytokine production, T-cell proliferation, and cytotoxicity against breast cancer cell line [128]. They showed that HER2-redirected CAR T cells including the 4-1BB costimulatory domain exerted more prominent tumor targeting with reduced T-cell exhaustion phenotype and ameliorated proliferative function than HER2-CARs containing the CD28 domain in vivo (109). Robustly, intracranial and also intraventricular administration of HER2-CAR T cells also elicited robust antitumor response in orthotopic xenograft models [128]. Besides, a novel humanized HER2-specific constructed by (1) CAR-containing chA21 scFv region of antigen-specific mAb and (2) intracellular signaling chains involving CD28 and CD3ζ abrogated tumor proliferation in human breast cancer SKBR3 cell tumor xenograft [129]. Further analysis indicated a potent capacity of human CD3-positive T cells in regressing SKBR3 lesions, delivering the proof of the concept that more exploration of the HER2-specific CAR T cell therapy for HER2-positive tumors can lead to more auspicious outcomes [129]. Similarly, the third-generation HER2-specific CAR T cells alone or in combination with anti-PD1 antibody suppressed the development of HER2-positive mouse breast tumor cells in vitro and tumor cell-bearing mice [130]. Based on the analysis, cytotoxicity was about 39% with CAR T cells alone and improved to 49% with using an anti-PD1 antibody [130]. In vivo, injected CAR T cells were detected in the tumor stroma, delayed tumor development, and also augmented tumor apoptosis [130]. In addition, bispecific switchable CAR T cells targeting both the HER2 and insulin-like growth factor 1 (IGF-1) receptor demonstrated a provoked activity against breast cells with low HER2 expression, documenting the CAR T cell’s capacity to eliminate tumor cells with low or heterogeneous HER2 expression [131]. Besides, Lenalidomide, a 4-amino-glutamyl analogue of thalidomide that acts as an angiogenesis inhibitor, showed the capability to intensify the HER2-CAR T cells cytotoxicity against human breast tumor MDA-MB-453 by induction higher levels of the cytokine secretion but not affecting their proliferation [132]. Moreover, lenalidomide induced a substantial decrease in Ikaros and Aiolos expression in HER2-CAR T cells, leading to the stimulated IL-2 secretion from T cells and augmenting their activities [132].

### Ovarian cancer

The natural history of ovarian cancer (OC) endures being described by late-stage presentation, metastatic bulky disorder burden, and stagnant mortality statistics [133]. The OC has no targeted molecular therapies for controlling its progress, especially resistant or relapsed OC. Preliminary studies have shown that targeting overexpressed molecules like mucin 16 (MUC16), annexin 2 (ANXA2), and also HER2 can sustain high tumor cells toxicity, and so dwindle tumor burden [134]. For instance, MUC16-redirected CAR T cells exhibited a therapeutic benefit versus human breast OVCAR-3 tumor-bearing mice and also prolonged their survival time [135]. Notwithstanding, because of the inherent heterogeneity of OC concurrently high mutation multiplicity and overexpression of diverse receptors, employing individual therapeutic strategies is largely desired [134]. It has been suggested that HER2-specific CAR T cells could stimulate robust and selective cytotoxicity against HER2 expressing established or primary ovarian cancer cells [136, 137]. In addition, bispecific antibodies (BsAbs) specific for CD20 or HER2 could ameliorate the cytolytic function of primary human T-cells against CD20-expressing leukemic cells or HER2-expressing epithelial cancer cells [136]. Also, HER2-specific CAR T cells could produce high levels of IFNγ following stimulation with SKOV3 cells [138]. Of course, tumor elimination mediated by anti-HER2 CAR T cells was accompanied by restored influx and expansion of the adoptively transferred CAR T cells [138]. As well, M1 macrophages and also IFNγ receptor expression on tumor stromal cells, but not NK cells, contributed to tumor lysis in tumor cell-bearing mice [138]. The achieved results imply that CAR T cell therapy is capable of eliminating solid tumors by a combination of antigen-independent stroma deterioration as well as antigen-specific tumor cell targeting [138]. Overall, HER2-specific CAR T cell therapy has exposed respectable therapeutic capability in the preclinical stage, while this intervention in OC is still in the clinical experimental phase.

### Gastrointestinal cancer

Gastrointestinal cancer refers to malignant circumstances of the gastrointestinal (GI) tract and other organs complicated in digestion, including the esophagus, stomach, biliary system, small intestine, large intestine, rectum, and anus. Studies on colorectal cancer (CRC) cell-bearing mice models have shown that HER2-specific CAR T cells hindered tumor progress in immunodeficient NOD-NPG mice [139]. Indeed, administration of HER2-redirected CAR T cells led to an abrogation or even eradication of CRC xenograft in the PDX model [139]. These appreciated events in turn resulted in an improved overall survival rate in transplanted mice [139]. Teng et al. [139] also observed that desired events might arise from the production of higher levels of IFN-γ as observed in the peripheral blood of CAR T cell recipients. These results indicated that HER2-redirected CAR T cells could show long-term persistence in vivo and efficiently remove the freshly implanted tumor tissues [139]. Besides, owing to this fact that innovative immunotherapeutic methods are instantly wanted for gastric cancer as its poor survival and unsatisfactory treatment, Han and his colleagues used the humanized chA21 scfv-based HER2-redirected CAR T cells to induced cytotoxicity against HER-2 expressing gastric cancers [140]. In vitro, the engineered chA21-4-1BBz CAR T cells elicited cytokine response along with effective elimination of HER2 expressing human gastric cancer cells [140]. They found that the cytokine release and cytotoxicity rates were in association with the level of HER2 expression by transformed cells. In vivo, chA21-4-1BBz CAR T cells intensely enabled cytolysis of HER2 overexpressing tumor and eventually expanded survival of tumor cell-bearing mice, while HER2 low-expressing tumor progressed [140]. These outcomes justified using the humanized chA21 scFv-based, 4-1BB costimulated CAR T cells for gastric cancer therapy [140]. Too, conventional HER2-specific CAR T cells also presented the remarkable capability to delay tumor progress, exert long-term survival, and display homing to targets in HER2-positive xenograft gastric tumors [141]. Also, it was proved that induction of the 4-1BB (CD137) pathway by an agonistic α-4-1BB antibody may offer durable costimulatory signals for enhancing T-cell responses [142]. In fact, α-4-1BB could considerably boost CAR T cell-triggered cytotoxicity against tumor cell-bearing mice [142]. Further, combination therapy could cause higher levels of expression of IFNγ and proliferation marker Ki67 in tumor-infiltrating CAR T cells [142]. Importantly, α-4-1BB could diminish host immunosuppressive cells at the tumor area, such as regulatory T (T regs) cells and myeloid-derived suppressor cells (MDSCs), and thereby could ameliorate therapeutic responses [142].

In addition to T cells, NK-92 cells engineered to express HER2-specific second-generation CAR (NK-92/5.137.z cells) selectively eradicated HER2-positive gastric cancer cells mediated possibly by higher levels of cytokine secretion in vitro [143]. In vivo, NK-92/5.137.z cells effectively eliminated small tumor xenografts, but not larger solid tumors [143]. Besides, treatment with apatinib, a well-known inhibitor of HER2, restored NK cell recruitment into large tumor xenografts and ultimately reinforced the therapeutic activities of NK-92/5.137.z cells [143].

### Others

Recent studies have indicated that HER2-redirected CAR T cells can eliminate uveal and cutaneous melanoma cells in vitro and in NOG mice [144]. Impaired anti-tumor activities of the CAR T cells following CRISPR/Cas9-mediated ablation of HER2 in the melanoma cells outlined the specific antitumor effects of HER2-specific CAR T cells against melanoma cells [144]. Moreover, HER2-redirected CAR T cells showed the competence to be noted as an effective and rational therapeutic option for refractory metastatic rhabdomyosarcoma, as documented in a child with metastatic rhabdomyosarcoma [145]. Also, studies have displayed that docetaxel (DOC), a chemotherapeutic agent, improved the infiltration of HER2-CAR T cells to tumor sites in the non-small-cell lung carcinoma (NSCLC) mice model [146]. DOC improved the expression of chemokine receptor-ligand C-X-C motif chemokine 11 (CXCL11) in TME, facilitating CD8+ T cell recruitment [146]. Remarkably, tumors from DOC-treated mice showed advanced expression of high-mobility group box 1 (HMGB1) and CXCL11, raised HER2-CAR T cell infiltration as well as tempered tumor progress [146]. Albeit, it has previously been suggested that HMGB1 plays a multifaceted role in tumor progress or therapy [147]. It enables CD8+ T cell recruitment to the tumor microenvironment, whereas HMGB1-stimulated autophagy may induce tumor resistance to chemotherapy [147]. In another report, Gao et al. promoted the potency, and duration of anti-tumor functions of HER2-redirected T cells employing an oncolytic virus (OV) that generated cytokine IL-12, checkpoint blockade, and a bispecific tumor-targeted T cell engager (BiTE) molecule, specific for CD44 variant 6 (CD44v6) [146]. The modified OVs were able to potentiate the capacity of HER2-redirected CAR T cells to eliminate multiple CD44v6+ cancer cell lines (human HNSCC line FaDu, human prostate cancer cell line PC-3, and human cervical cancer cell line SiHa). These OVs also instigated more speedy and continued tumor control of orthotopic HER2+ and HER2−/− CD44v6+ tumors than monotherapies with HER-2 CAR T cell or modified OVs [146]. Interestingly, a phase I/II clinical study in 19 participants suffering from recurrent/refractory HER2-positive sarcoma was carried out by Ahmed et al. [148]. During follow-up, the injected cells were well tolerated without any dose-limiting toxicity. HER2-redirected CAR T cells persisted for about 6 weeks in 7 of the 9 evaluable patients and also were identified at tumor sites of 2 patients [148]. Finally, of 17 evaluable patients, 4 experienced stable disease for approximately 3 months, while 3 of them showed mitigated tumor progress [148]. This study for the first time indicated that HER2-CAR T cell therapy can be safe and effective, and also can persist for 6 weeks without serious untoward effects in patients with tumors [148].

### The application of genome-editing tools in CAR-T cell therapies

As described, the genome-editing tools underlie a paradigm shift in immune cell-based tumor therapies. Irrespective of the offering the possibility for manufacturing allogeneic T cells, these technologies support establishing the next generation of CAR T cells with higher efficacy, circumventing immune-suppressive TME. Remarkably, PD-1 ablation results ultimately in producing CAR T cells with higher resistance to PDL-1 expressed on the surface of tumor cells [149]. For instance, PD-1-knocked out anti-EGFRvIII CAR T cells could stimulate the elimination of EGFRvIII expressing glioblastoma cells more prominently than conventional anti-EGFRvIII CAR T cells [149]. Similarly, PD-1-knocked out CD19-redirected CAR T cells displayed more evident anti-tumor function versus CD19+PD-L1+K562 leukemic cells in vitro and in NSG mice [150]. Further, PD-1 ablation by the CRISPR tool provides CAR T cells which higher cytotoxicity against mesothelin-expressing triple-negative breast cancer (TNBC) [151]. On the other hand, lymphocyte activation gene-3 (LAG-3) deficient anti-CD19 CAR T cells potently induced robust antigen-specific anti-leukemia impacts in vitro and in NPG mice [152]. Other studies have shown that transforming growth factor beta receptor (TGF-βR) ablation using genome-editing tools raised prostate-specific membrane antigen (PSMA)-specific CAR T cell expansion and powerfully motivated PSMA-expressing prostate cancer eradication in vitro. These modified cells also could display boosted cytokine release, resistance to exhaustion, and also extended persistence in vivo [153]. Moreover, the blockade of TGFBR2 expression in mesothelin-specific CAR T cells by CRISPR/Cas9 technique reduced the functional Treg conversion and prohibited CAR T cells depletion [154]. These cells could trigger increased cytotoxicity toward B-cell maturation antigen (BCMA)-positive myeloma cells than conventional CAR T cells in the presence of TGFβ in TME [155]. Undoubtedly, combining CAR technologies with genome-targeting tools can deliver immune cell’s higher anti-tumor responses in vitro and in vivo, providing a valued opportunity for human tumor therapies with better efficacy.

## Conclusion and future direction

**Fig. 2:**
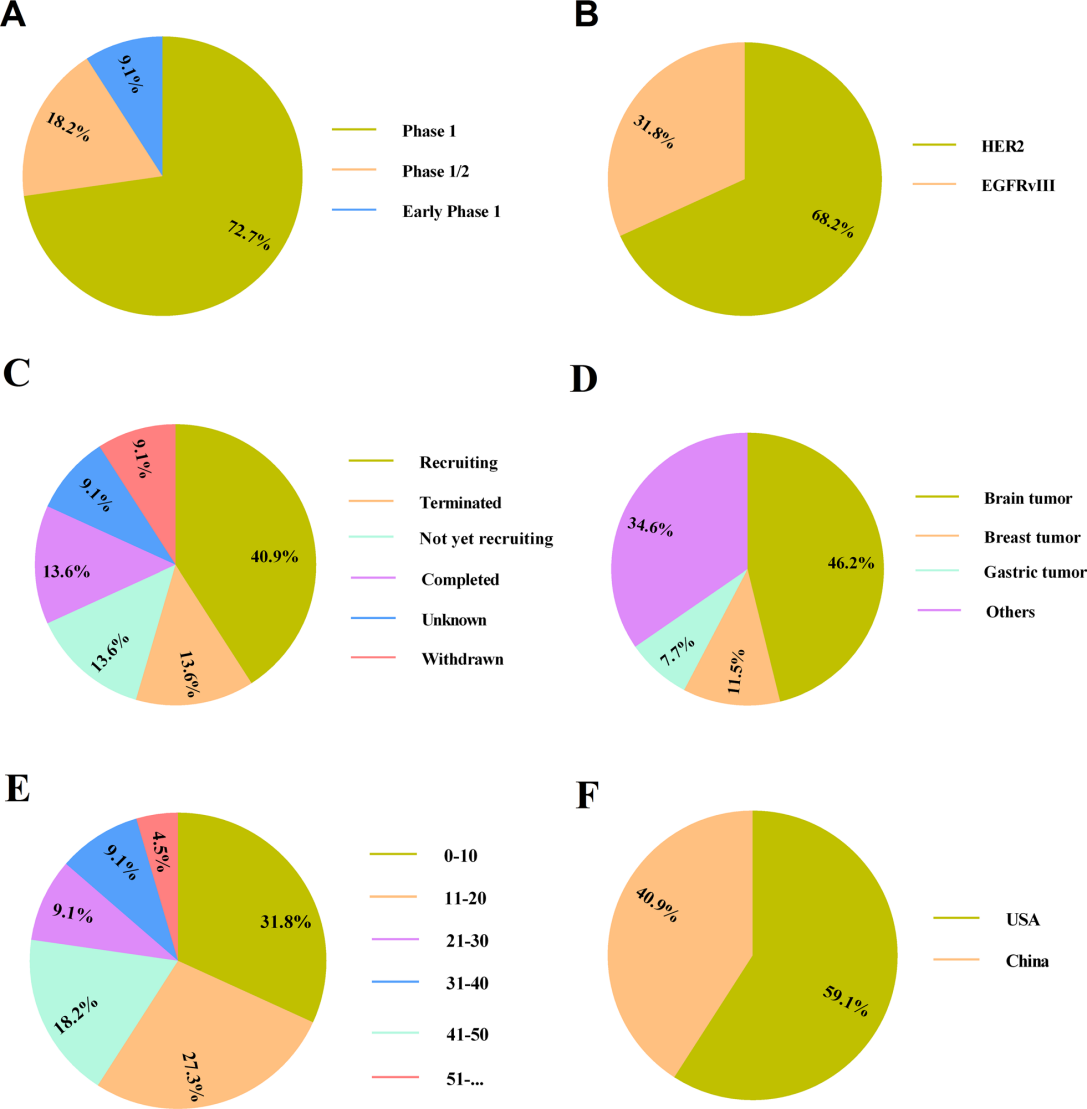
Clinical trials based on targeting EGFR family members, especially HER2, using human CAR T cells registered in ClinicalTrials.gov (September 2021). The schematic presents clinical trials respecting the treatment of EGFR-positive tumors using CAR T cells depending on the study phase (**A**), target antigen (**B**), study status (**C**), tumors (**D**), participant number (**E**), and study location (**F**). Human epidermal growth factor receptor 2 (HER2), Epidermal growth factor receptor (EGFR)

Recently, CAR T and also CAR NK cell-based therapy has become a game-changing tool in the context of tumor immunotherapies. Meanwhile, HER2-redirected CAR T or NK cells have demonstrated strong antitumor effects in preclinical reports (Tables 2, 3), as well as have shown remarkable safety and modest efficacy in clinical trials (Fig. 2, Table 4). However, on-target off-tumor toxicity obstructs the clinical utility of CAR T cells for solid tumors therapy. Thereby, we think that a special focus on improving the CAR-redirected immune cell safety and efficacy and also preparing universal redirected cells must be taken. Also, thanks to the occurrence of higher rates of CRS following CAR T cell therapy, it seems that development of novel generations of CAR and also more comprehensive focus on evolving CAR NK cell is urgently required. Further, the expression of PD1 as an immunosuppressive molecule expressed on the surface of activated T cells leads largely to the tumor cell resistance to CAR T cell-elicited cytotoxicity. Now, much effort has been spent to combine genome-editing technologies with CAR T cell therapies. Indeed, ablation of PD-1 by gene editing tolls can offer CAR T cell with higher safety and efficacy. Importantly, as CAR T cell-derived exosomes do not express PD1, and thereby their anti-tumor impact cannot be abrogated by PD-L1 in TME, using CAR T cell derived exosome has become an innovative therapeutic plans to potentiate therapeutic merits of this therapeutic modality.

**Table 4:** A summary of clinical trials based on the administration of CAR T cells to target EGFR, in particular HER2, -positive tumor cells registered in ClinicalTrails.gov (September 2021)

Condition	CAR- immune cell	Study phase	Status	Participant number	Location	NCT number
Glioma	EGFR-vIII CAR-T	1	Terminated	11	USA	NCT02209376
Glioma	EGFR-vIII CAR-T	1	Terminated	2	USA	NCT03283631
Glioma	EGFR-vIII CAR-T	1	Terminated	3	USA	NCT02664363
Central nervous system tumor	HER2 CAR-T	1	Recruiting	48	USA	NCT03500991
Brain tumor Breast cancer	HER2 CAR-T	1	Recruiting	39	USA	NCT03696030
Glioma	EGFR-vIII CAR-T	1/2	Completed	18	USA	NCT01454596
Various cancers	HER2 CAR-T	1/2	Withdrawn	0	China	NCT02713984
Lung cancer	HER2 CAR-T	1	Recruiting	30	China	NCT03198052
Breast cancer Gastric cancer	HER2 CAR-T	1	Recruiting	220	USA	NCT04650451
Glioma	EGFR-vIII CAR-T	1	Unknown	20	China	NCT02844062
Central nervous system tumor	HER2 CAR-T	1	Recruiting	28	USA	NCT02442297
Ependymoma	HER2 CAR-T	1	Not yet recruiting	50	USA	NCT04903080
Various cancers	HER2 CAR-T	1	Recruiting	45	USA	NCT03740256
Glioma	EGFR-vIII CAR-T	1	Completed	7	USA	NCT03726515
Various cancers	HER2 CAR-T	1	Recruiting	18	USA	NCT04660929
Glioma	HER2 CAR-T	1	Completed	16	USA	NCT01109095
Peritoneal carcinoma metastatic	HER2 CAR-T	Early 1	Not yet recruiting	18	China	NCT04684459
Various cancers	HER2 CAR-T	1	Recruiting	15	China	NCT04511871
Sarcoma	HER2 CAR-T	1	Active, not recruiting	36	USA	NCT00902044
Esophagus cancer Hepatoma Glioma Gastric cancer	EGFR-vIII CAR-T	1/2	Recruiting	50	China	NCT03941626
Breast cancer	HER2 CAR-T	1/2	Withdrawn	0	China	NCT02547961
Pancreatic cancer	HER2 CAR-T	Early1	Unknown	10	China	NCT03267173

*NA* not applicable

## Data Availability

Not applicable.

## Abbreviations

HER2: Human epidermal growth factor receptor 2
CAR: Chimeric antigen receptor
EGFR: Epidermal growth factor receptor
TME: Tumor microenvironment
NK cells: Natural killer cells
PB: Peripheral blood
BM: Bone marrow
TAAs: Tumor-associated antigens
CRS: Cytokine release syndrome
GVHD: Graft-versus-host disease
CRISPR: Clustered regularly interspaced short palindromic repeat
PD-1: Programmed cell death protein 1
